# Surface α-1,3-Glucan Facilitates Fungal Stealth Infection by Interfering with Innate Immunity in Plants

**DOI:** 10.1371/journal.ppat.1002882

**Published:** 2012-08-23

**Authors:** Takashi Fujikawa, Ayumu Sakaguchi, Yoko Nishizawa, Yusuke Kouzai, Eiichi Minami, Shigekazu Yano, Hironori Koga, Tetsuo Meshi, Marie Nishimura

**Affiliations:** 1 National Institute of Agrobiological Sciences (NIAS), Tsukuba, Ibaraki, Japan; 2 Ritsumeikan University, Kusatsu, Japan; 3 Department of Bioproduction Science, Ishikawa Prefectural University, Ishikawa, Japan; University of Melbourne, Australia

## Abstract

Plants evoke innate immunity against microbial challenges upon recognition of pathogen-associated molecular patterns (PAMPs), such as fungal cell wall chitin. Nevertheless, pathogens may circumvent the host PAMP-triggered immunity. We previously reported that the ascomycete *Magnaporthe oryzae*, a famine-causing rice pathogen, masks cell wall surfaces with α-1,3-glucan during invasion. Here, we show that the surface α-1,3-glucan is indispensable for the successful infection of the fungus by interfering with the plant's defense mechanisms. The α-1,3-glucan synthase gene *MgAGS1* was not essential for infectious structure development but was required for infection in *M. oryzae*. Lack or degradation of surface α-1,3-glucan increased fungal susceptibility towards chitinase, suggesting the protective role of α-1,3-glucan against plants' antifungal enzymes during infection. Furthermore, rice plants secreting bacterial α-1,3-glucanase (AGL-rice) showed strong resistance not only to *M. oryzae* but also to the phylogenetically distant ascomycete *Cochlioborus miyabeanus* and the polyphagous basidiomycete *Rhizoctonia solani*; the histocytochemical analysis of the latter two revealed that α-1,3-glucan also concealed cell wall chitin in an infection-specific manner. Treatment with α-1,3-glucanase *in vitro* caused fragmentation of infectious hyphae in *R. solani* but not in *M. oryzae* or *C. miyabeanus*, indicating that α-1,3-glucan is also involved in maintaining infectious structures in some fungi. Importantly, rapid defense responses were evoked (a few hours after inoculation) in the AGL-rice inoculated with *M. oryzae*, *C. miyabeanus* and *R. solani* as well as in non-transgenic rice inoculated with the *ags1* mutant. Taken together, our results suggest that α-1,3-glucan protected the fungal cell wall from degradative enzymes secreted by plants even from the pre-penetration stage and interfered with the release of PAMPs to delay innate immune defense responses. Because α-1,3-glucan is nondegradable in plants, it is reasonable that many fungal plant pathogens utilize α-1,3-glucan in the innate immune evasion mechanism and some in maintaining the structures.

## Author Summary

*Magnaporthe oryzae*, *Cochlioborus miyabeanus*, and *Rhizoctonia solani* are the top three fungal pathogens that are responsible for devastating damage to the production of rice, a staple cereal for half of the world's population. These fungal pathogens infect host plants despite the plants' innate immunity, which is activated upon recognition of a conserved cell wall component in fungi, such as chitin. Fungal pathogens seem to have evading mechanism(s) against the host innate immunity; however, the mechanisms are still unclear. In this study, we discovered a novel mechanism that is commonly used by fungal pathogens to prevent host innate immunity. In this mechanism, fungal pathogens mask the cell wall surfaces with α-1,3-glucan, a polysaccharide that plants cannot degrade. In fact, a transgenic rice secreting a bacterial α-1,3-glucanase, which is able to remove α-1,3-glucan on the fungal surfaces, obtained strong resistance to all of those fungal pathogens. We also showed that plants rapidly activated defense responses against fungi (even before the fungal penetration) when α-1,3-glucan on the fungal surfaces were damaged or removed. Our study suggests that fungal surface α-1,3-glucan interferes with host immunity in many fungal pathogens and that α-1,3-glucan is a potential target for controlling various fungal diseases in plants.

## Introduction

The fungal cell wall, the outermost layer of the cell, is a physically rigid structure responsible for protecting the cell from environmental stresses and maintaining cell morphology. The fungal cell wall is primarily composed of polysaccharides, such as β- and α-glucans, chitin and mannans; however, the spatial organization of polysaccharides has only been partially solved [1], [2]. In general, the structural core of the fungal cell wall is composed of branched β-1,3-glucan cross-linked to chitin. This core complex is generally fibrillar and embedded in alkali-soluble polysaccharides that vary according to fungal species, growth stages and environments [1], [2]. In plant-fungus interactions, plants recognize fungus-specific molecules, such as cell wall polysaccharides, cell membrane sterols and secreted proteins, as pathogen-associated molecular patterns (PAMPs) via pattern recognition receptors (PRRs) that initiate immediate innate immune responses, including the production of antifungal enzymes, anti-microbial metabolites and reactive-oxygen species, against a broad range of fungal invaders [3]–[7]. The fungal cell wall polysaccharides are primary targets of PRR recognition because they are displayed on fungal surfaces and conserved in a broad range of fungi. However, fungal pathogens are able to infect host plants; therefore, they are expected to acquire some mechanisms to escape the recognition of their cell wall PAMPs by the host PRRs.

*Magnaporthe oryzae* is an ascomycete monocot pathogen that causes rice blast, which is the most serious disease in global rice production [8]. Under natural conditions, *M. oryzae* produces an appressorium, which is a dome-shaped infection-specific structure, at the tip of the germ tube extending from the conidium on the plant cuticle. A penetration peg developed from the appressorium pierces through the host plant cuticle and subsequently differentiates into infectious hyphae [8], [9]. Although the cell wall of *M. oryzae* contains chitin [10], [11], a PAMP recognized by rice PRR [12], [13], the fungus is capable of circumventing innate immune recognition in rice. In a previous study using histocytochemistry, we revealed that *M. oryzae* responds to a plant wax component and accumulates α-1,3-glucan on the surface of the cell wall of infectious structures [10]. Consequently, α-1,3-glucan present on the surface of the fungus masks the chitin and β-1,3-glucan in the cell wall and interferes with the enzymatic digestion of chitin *in vitro* [10].

In this report, we showed that the surface-accumulated α-1,3-glucan in *M. oryzae* is essential for successful infection. Lack of α-1,3-glucan resulted in rapid activation of host defense responses, indicating its role in protection against antifungal agents secreted by plants and evasion from rice PRR recognition. The surface α-1,3-glucan is also required for the successful infection of rice by the ascomycete *Cochlioborus miyabeanus* and the polyphagous basidiomycete *Rhizoctonia solani*, which are phylogenetically distant from *M. oryzae*. There are no annotated α-1,3-glucanase genes in plant genomes; therefore, our results suggest that masking the cell wall surface with α-1,3-glucan is a stealth infection strategy of fungal plant pathogens. Moreover, our study provides novel plant protection approaches that target fungal α-1,3-glucan to control a broad spectrum of fungal diseases.

## Results

### Surface α-1,3-glucan is necessary for live plant infection in *M. oryzae*

**Figure 1:**
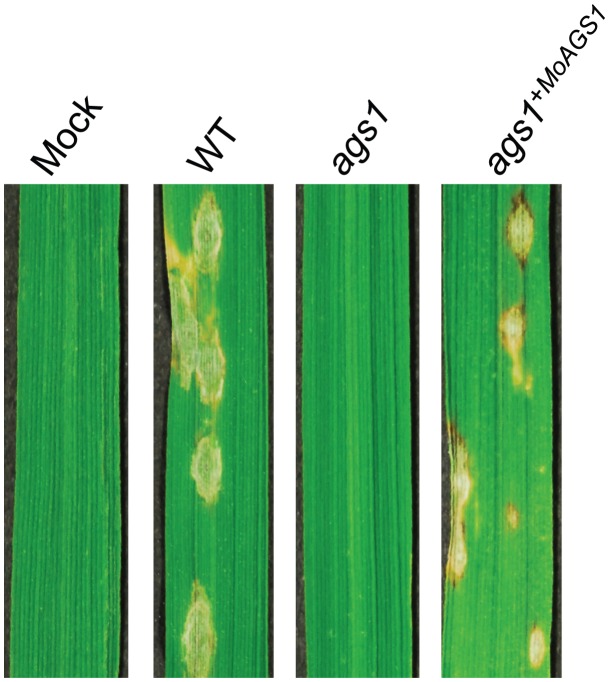
The loss of pathogenicity in *ags1*. Susceptible rice leaves were spray-inoculated with the *M. oryzae* wild-type (WT), *ags1*, or *ags1* ^+*MoAGS1*^ strains or with distilled water (mock). Typical blast-disease lesions were observed on the rice leaves inoculated with WT and *ags1* ^+*MoAGS1*^, but the lesions were barely observed on the leaves inoculated with *ags1*. The assays were repeated at least 10 times. The photos were taken at 7 days post inoculation (dpi).

Based on database searches, which did not reveal any genes encoding α-1,3-glucanase in the rice genome, we speculated that the surface α-1,3-glucan might play an important role in protecting *M. oryzae* during infection of the host. To clarify the role of the surface-accumulated α-1,3-glucan in fungal infection, we generated an *M. oryzae* mutant, *ags1*, lacking the α-1,3-glucan synthase gene *MoAGS1* (Figure S1). Compared with the wild-type strain, *ags1* exhibited a comparable level of mycelial growth. The conidiation in *ags1* was reduced to approximately 30% of that observed in the wild-type strain, but there were no defects in appressorium formation on the inductive surfaces or in the development of infectious hyphae in cells of heat-killed rice or onion epidermis (Figure S2, Table S1). Histocytochemical analyses showed that *ags1* lacked α-1,3-glucan and thereby β-1,3-glucan and chitin were exposed, in contrast to the wild-type strain in which β-1,3-glucan and chitin were undetectable due to α-1,3-glucan masking [10] (Figure S2B). Spray-inoculation of *ags1* did not induce formation of lesions on the rice leaves, whereas the inoculation of either the wild-type or *ags1* ^+*MoAGS1*^ strain induced the development of many lesions (Figure 1).

**Figure 2:**
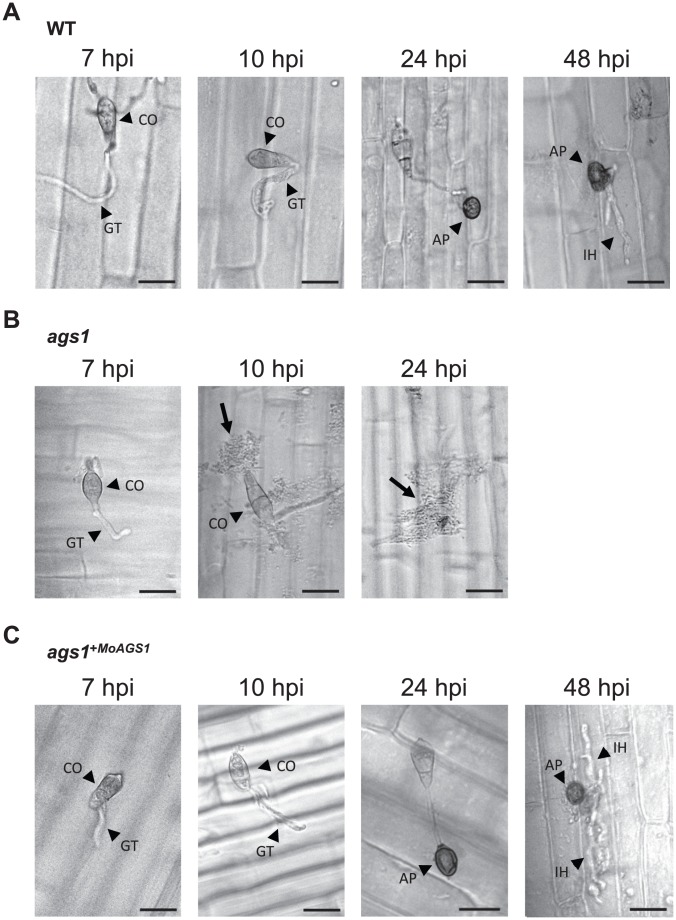
The histocytological observation of the infectious fungal structure development of *ags1*. The *M. oryzae* strains were inoculated on the live leaf sheath cells of the susceptible rice cultivar LTH. (**A**) WT, (**B**) *ags1* and (**C**) *ags1* ^+*MoAGS1*^. The appressoria and infectious hyphae were observed at 24 hpi and 48 hpi, respectively, for both WT and *ags1* ^+*MoAGS1*^. The destruction of *ags1* (indicated by arrows) appeared at 10 hpi and was apparent at 24 hpi. The arrowheads indicate conidium (CO), germ tube (GT), appressorium (AP), and infectious hyphae (IH). Scale bar = 20 µm. More than 50 conidia were observed at one time and this observation was repeated 10 times. Representative images are presented.

We further examined the development of infectious structures on live susceptible rice sheath cells. In both the wild-type and *ags1* ^+*MoAGS1*^ strains, the differentiation of the appressoria on the tips of germ tubes was observed at 24 h post inoculation (hpi), followed by the development of infectious hyphae in rice cells at 48 hpi (Figure 2A and 2C). Strikingly, most of *ags1* was destroyed before penetration into the plant (Figure 2B, 24 hpi). The destruction of *ags1* prior to penetration and the loss of pathogenicity were also observed in the susceptible live barley plants (Figure S3).

Because the surface-accumulated α-1,3-glucan increased the tolerance of the cell wall to chitinase in *M. oryzae* [10], *ags1* was expected to be more sensitive to chitinase treatment than the wild type. As expected, >60% of the *ags1*germ tubes were lysed by chitinase alone under the condition where approximately 10% of the wild-type and *ags1* ^+*MoAGS1*^ germ tubes were lysed (Figure S4). In contrast, treatment with enzyme mixture of chitinase and α-1,3-glucanase increased the ratio of the lysed germ tubes for the wild type and *ags1* ^+*MoAGS1*^ but not for *ags1* (Figure S4). Together with our observation that α-1,3-glucan was not required for infectious structure development but was indispensable for live host plant infection in *M. oryzae* (Figures 1, S2 and S3, and Table S1), α-1,3-glucan is suggested to be involved in the protection of the fungal cell wall against digestive enzymes secreted by plants during infection.

### An *M. oryzae* mutant lacking surface α-1,3-glucan rapidly activated defense responses in susceptible host plants

**Figure 3:**
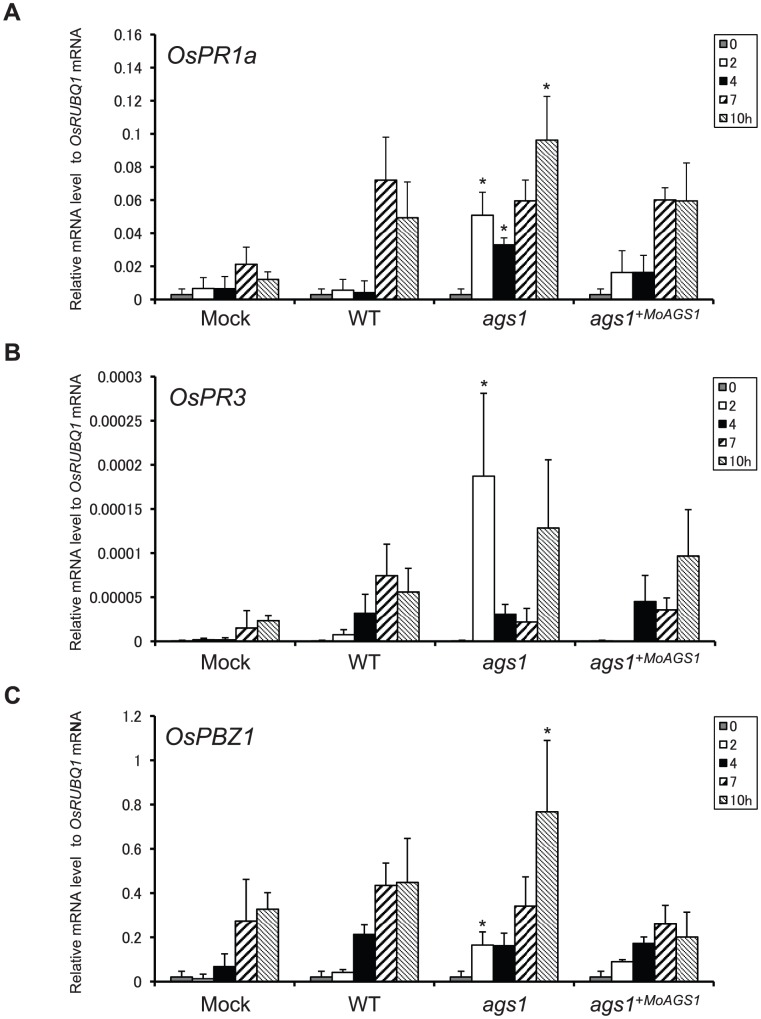
The expression of representative PR genes in rice inoculated with *M. oryzae*. (**A**) *OsPR1a*, (**B**) *OsPR3* and (**C**) *PBZ1*. Rice plants were inoculated with distilled water (mock), WT, *ags1*, or *ags1* ^+*MoAGS1*^ *M. oryzae* strains. The gene expression levels relative to the *OsRUBQ1* gene were analyzed using qRT-PCR at 0, 2, 4, 7, and 10 hpi. The expression of the PR genes was rapidly induced through *ags1* inoculation. The results represent the mean ± SD from at least three independent experiments. An asterisk indicates statistically significant differences against the control (mock) at the same time points using the Dunnett's test at *P*<0.05.

Because *ags1* appeared to be sensitive to digestive enzymes present in the rice cell wall due to the lack of surface α-1,3-glucan, PAMPs could be released at an earlier time after infection by *ags1* compared to the wild type infection. To know whether the lack of α-1,3-glucan evoked defense responses more rapidly in rice, we analyzed expression levels of the pathogenesis-related protein (PR) genes in rice challenged by *ags1*. The expression of the PR genes *OsPR1a*, *OsPR3* (encoding a chitinase) and *PBZ1* (*OsPR10a*), which are indicators of rice defense responses [14], was significantly upregulated in the *ags1*-inoculated leaf sheaths (Figure 3). Notably, the expression of *OsPR1a* and *OsPR3* was rapidly induced; their induction was observed at 2 hpi with *ags1*, which was approximately 6 h before the appearance of incipient appressoria (Figure 3A and 3B). Thus, the surface α-1,3-glucan plays an important role in delaying host defense responses.

### Transgenic rice plants expressing bacterial α-1,3-glucanase activated defense responses in plants toward a compatible *M. oryzae* strain

Because the surface α-1,3-glucan is indispensable for successful fungal infection, we tested whether the expression of an α-1,3-glucanase gene in rice could quickly activate defense responses upon infection with *M. oryzae*. We generated transgenic rice plants that constitutively express the *Bacillus circulans* α-1,3-glucanase gene (*AGL*) [15] under the control of an 35S promoter (Figure S5A). The expression of the *AGL* gene and the Agl protein were confirmed (Figure S5B and S5C), and the Agl-expressing rice (AGL-rice) grew similarly to non-transgenic plants under our greenhouse conditions (Figure S6).

**Figure 4:**
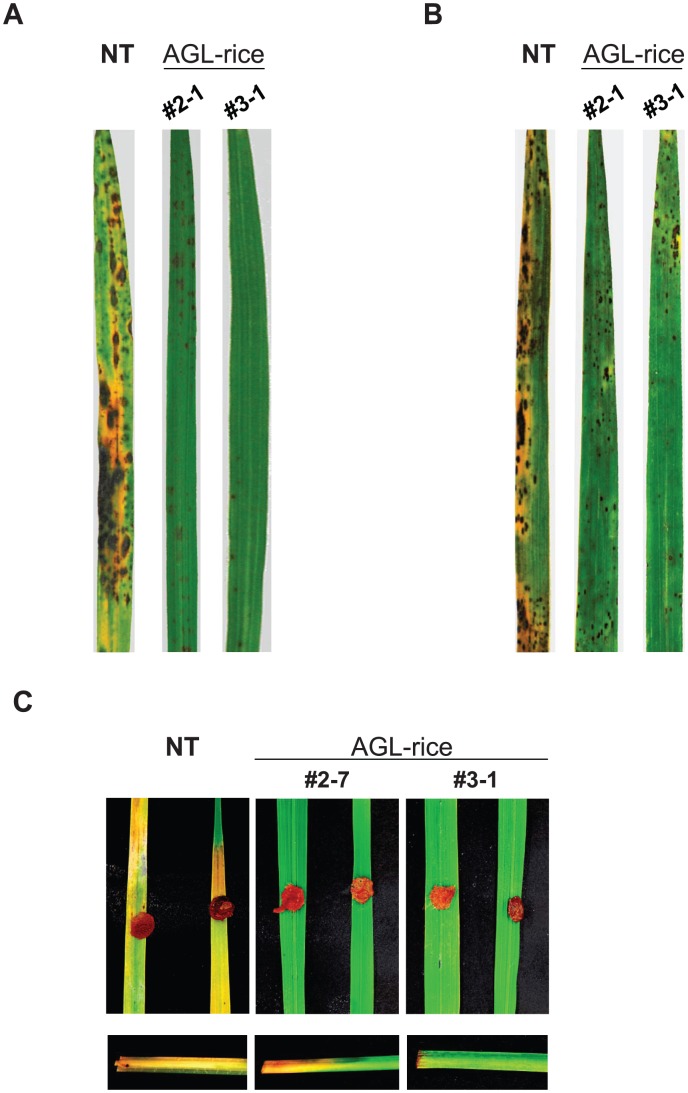
The fungal disease resistance of the transgenic rice expressing a bacterial α-1,3-glucanase (AGL-rice). (**A**) Spray-inoculation assay of *M. oryzae*. Typical blast-disease lesions were observed on the non-transgenic (NT) leaves. In contrast, HR-like cell death was observed with the AGL-rice leaves (#2-1 and #3-1). The assay was repeated 20 times, and representative images are presented. The photos were taken at 6 dpi. (**B**) Spray-inoculation assay of *Cochliobolus miyabeanus*. Typical brown spot lesions were formed and coalesced on the NT rice leaves, while the symptoms were significantly decreased on the AGL-rice leaves. The assay was repeated 20 times, and representative images are presented. The photos were taken at 6 dpi. (**C**) Inoculation assay of *Rhizoctonia solani*. Rot was observed on the NT rice leaves, but not on the AGL-rice leaves. The rotted region on the culms was greatly reduced in the AGL-rice compared with the NT rice. Each assay was repeated 10 times, and representative images are presented. The photos were taken at 6 dpi.

**Figure 5:**
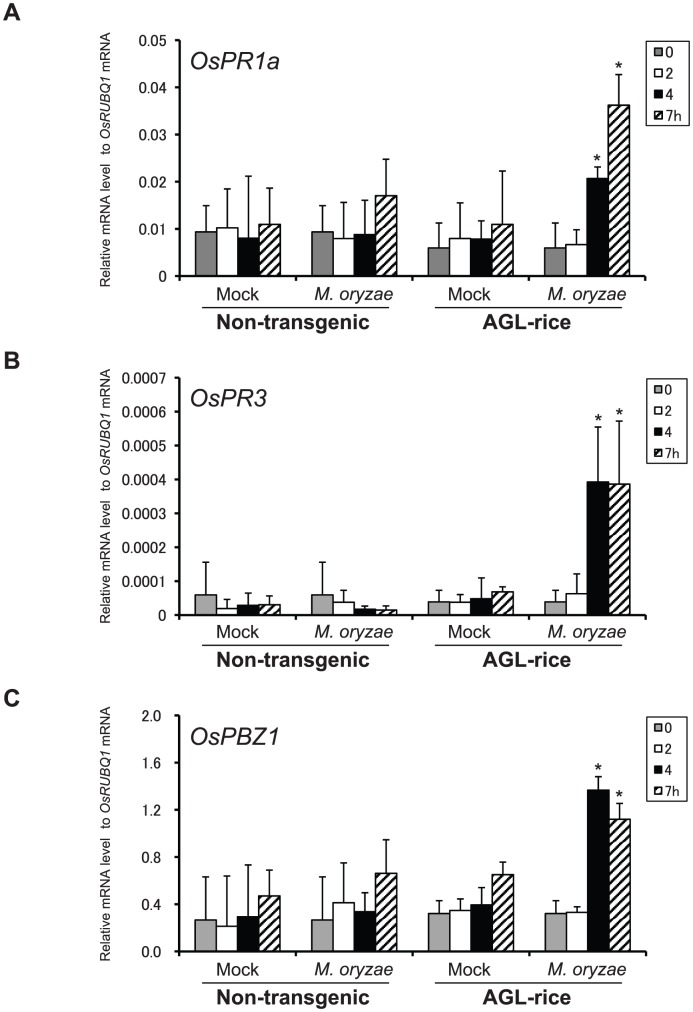
The expression of representative PR genes in *AGL*-expressing rice (AGL-rice) inoculated with *M. oryzae*. The expression of (**A**) *OsPR1a*, (**B**) *OsPR3* and (**C**) *PBZ1* relative to the *OsRUBQ1* gene. Rice plants were sprayed with a wild-type *M. oryzae* (Ina86-137) conidial suspension or distilled water (mock). The gene expression was analyzed using qRT-PCR at 0, 2, 4, and 7 hpi. The results represent the mean ± SD from at least three independent experiments. The significant differences against the control (mock) at the same time point in each series of tests are indicated by asterisks (Dunnett's test, *P*<0.05).

When inoculated with a compatible *M. oryzae* strain, the AGL-rice plants exhibited a highly resistant phenotype; only a few HR (hypersensitive response)-like necrotic spots were observed on the AGL-rice plants, whereas many lesions developed on the non-transgenic plants (Figure 4A). Consistent with these findings, the expression of the PR genes *OsPR1a*, *OsPR3*, and *PBZ1* was markedly induced as early as 4 hpi in the AGL-rice that had been spray-inoculated with *M. oryzae* conidia, but not in either the mock-inoculated AGL-rice or the mock-inoculated or *M. oryzae*-inoculated non-transgenic rice (Figure 5). Thus, the AGL-rice acquired the ability to activate a defense response upon fungal challenge, that was otherwise suppressed in the non-transgenic plants.

To determine the stage at which the infection of *M. oryzae* was inhibited, we examined the development of the fungal infectious structures on the leaf sheath cells of AGL-rice. At 48 hpi, approximately 85% of *M. oryzae* conidia produced appressoria on the non-transgenic rice but only 25% on the AGL-rice. In addition, only 20% of appressorium-forming conidia developed infectious hyphae in the AGL-rice cells, while approximately 85% developed infectious hyphae in the non-transgenic rice cells (Figure S7). Thus, the AGL-rice was able to inhibit fungal infection before cellular invasion. Because the *in vitro* treatment with α-1,3-glucanase did not prevent the development of infectious structures (Figure S8), the breakdown of surface α-1,3-glucan on the fungal cell wall appeared to make the fungi more sensitive against the antifungal agents secreted by plants. Consistent with this idea, α-1,3-glucanase activity was detected not only in the total protein fraction (7.52±0.97 mg released glucose·h^−1^·mg^−1^ protein) but also in the extracellular (apoplastic and cell wall) protein fraction (17.83±4.11 mg released glucose·h^−1^·mg^−1^ protein) extracted from the culms and leaf sheaths of the AGL-rice. No α-1,3-glucanase activity was detected in the total or extracellular protein fractions derived from the non-transgenic rice. Our results suggest that the AGL-rice activated defense responses against *M. oryzae* prior to fungal invasion into the cells, which further supports the idea that the surface-accumulated α-1,3-glucan protects the fungal cells from antifungal agents and also delays the release of PAMPs.

### Surface α-1,3-glucan is required for successful infection in multiple fungal pathogens

Similar to *M. oryzae*, other fungal pathogens could potentially conceal their cell wall PAMPs with α-1,3-glucan to escape from the innate immune defenses of the host plants. We investigated whether the AGL-rice was resistant to other economically important fungal pathogens of rice whose corresponding resistance genes have not been identified [16], such as *Cochliobolus miyabeanus*, an ascomycete that causes brown spots, and *Rhizoctonia solani*, a polyphagous basidiomycete that causes sheath blight [17]. Importantly, the AGL-rice showed strong resistance to both pathogens (Figure 4B and 4C). Small HR-like necrotic spots were dispersed on the AGL-rice leaves upon *C. miyabeanus* inoculation, whereas many enlarged and coalesced lesions, which eventually killed the leaves, were present on the leaves of non-transgenic rice under the same conditions (Figure 4B). In addition, the AGL-rice leaves showed no disease symptoms after *R. solani* inoculation, but the non-transgenic leaves were wilted and yellow (Figure 4C, upper panel). The rotted regions were more extensive on the culms of the non-transgenic rice than on those of the AGL-rice upon *R. solani* inoculation at one end of the culm (Figure 4C, lower panels). Thus, the expression of α-1,3-glucanase in rice plants confers strong resistance against multiple fungal pathogens.

**Figure 6:**
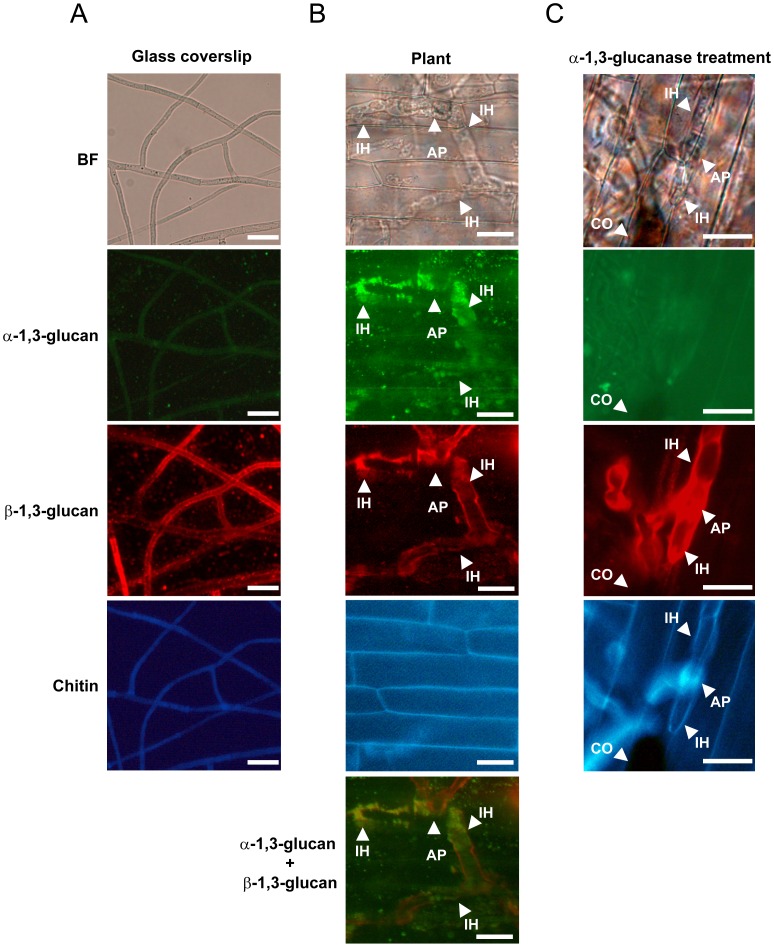
The detection of polysaccharides on the hyphal cell walls of *C. miyabeanus*. α- and β-1,3-glucan were detected using fluorophore-labeled antibodies, and chitin was detected using a fluorophore-labeled lectin. (**A**) The detection of polysaccharides at the accessible surface of the cell wall of mycelia developed on glass coverslips. Representative images from more than 100 fungal cells from two independent experiments are presented. (**B**) The detection of polysaccharides at the accessible surface of the cell wall of infectious hyphae developed on rice sheath cells. Layered images of α- and β-1,3-glucan are presented as ‘α-1,3-glucan+β-1,3-glucan’. Representative images of 50 fungal cells from 10 independent experiments are presented. (**C**) The detection of polysaccharides in the cell wall of infectious hyphae treated with α-1,3-glucanase. The representative images of 50 fungal cells from 10 independent experiments are presented. BF, bright-field optics; AP, appressorium; IH, infectious hyphae; CO, conidia. Scale bar = 20 µm.

**Figure 7:**
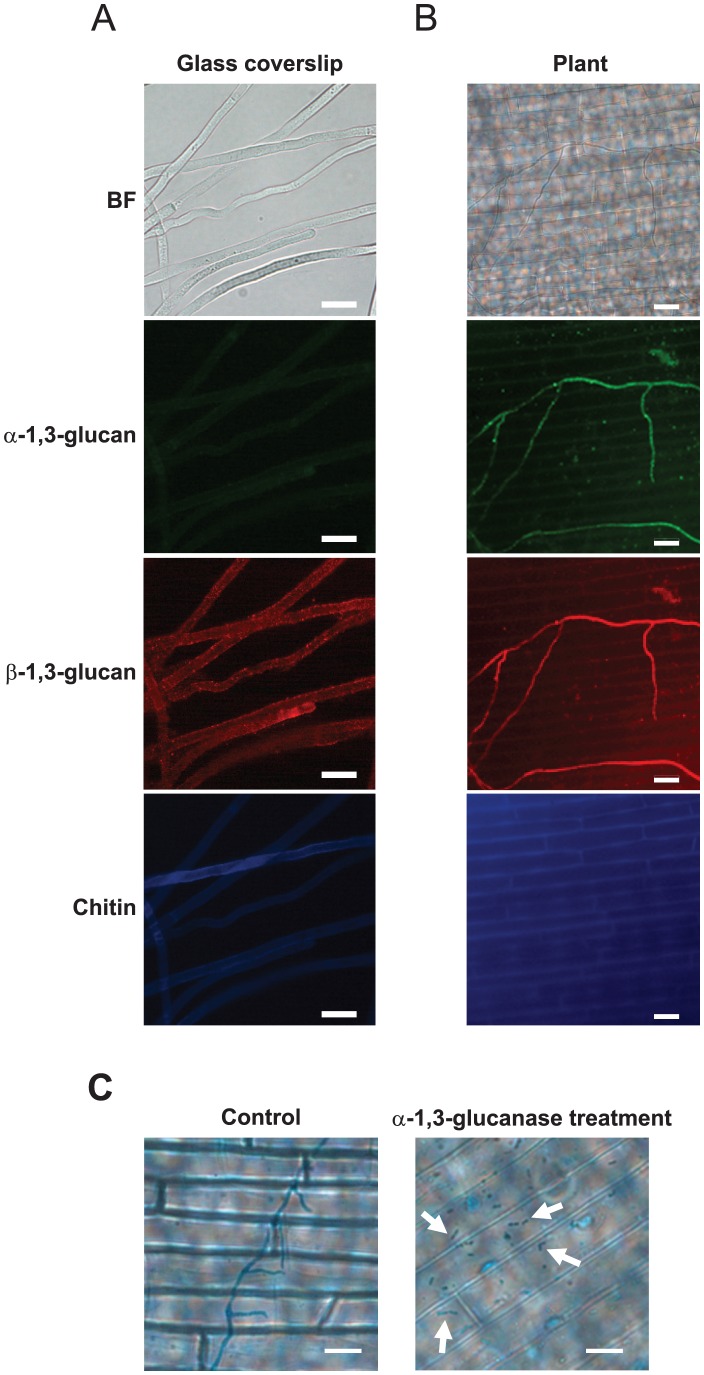
Detection of polysaccharides on the cell walls of *R. solani* hyphae. α- and β-1,3-glucan were detected using fluorophore-labeled antibodies, and chitin was detected using a fluorophore-labeled lectin. (**A**) The detection of polysaccharides at the accessible surface of the cell wall of mycelia developed on glass coverslips. Representative images of 50 fungal cells from 10 independent experiments are presented. (**B**) The detection of polysaccharides at the accessible surface of the cell wall of infectious hyphae developed on rice sheath cells. The representative images of more than 150 mycelia from 15 independent experiments are presented. (**C**) The degradation of infectious hyphae after treatment with α-1,3-glucanase. The infectious hyphae developed on more than 15 rice sheaths from 5 rice plants were stained with lactophenol cotton blue and observed. BF: bright-field optics. Scale bar = 20 µm.

To examine whether α-1,3-glucan inducibly accumulated on *C. miyabeanus* and *R. solani* during infection similarly to *M. oryzae*, we histocytochemically examined the localization of α-1,3-glucan in the cell wall of the fungal hyphae. In *C. miyabeanus* and *R. solani*, chitin and β-1,3-glucan were detected at the accessible surface of the cell wall of mycelia developed on glass coverslips, whereas α-1,3-glucan was barely detected (Figure 6A and 7A). In contrast, α-1,3-glucan and β-1,3-glucan, but not chitin, were clearly detected on the accessible surface of the cell wall of the infectious hyphae in heat-killed rice cells (Figure 6B and 7B). Thus, α-1,3-glucan is induced to accumulate at the surface of the hyphal cell wall in an infection-specific manner, which is common among these rice fungal pathogens. Notably, the enzymatic digestion of α-1,3-glucan revealed an underlying chitin in the cell wall of infectious hyphae in *C. miyabeanus* (Figure 6C) and resulted in the fragmentation of the hyphae in *R. solani* (Figure 7C). In *C. miyabeanus*, the *in vitro* treatment with α-1,3-glucanase did not affect the development of the infectious structure (Figure S8). Taken together, these results indicate that, similar to *M. oryzae*, α-1,3-glucan masks chitin in the cell wall of infectious hyphae in *C. miyabeanus* and *R. solani*. Moreover, in *R. solani*, α-1,3-glucan is essential for the maintenance of the infection-specific hyphal structure.

**Figure 8:**
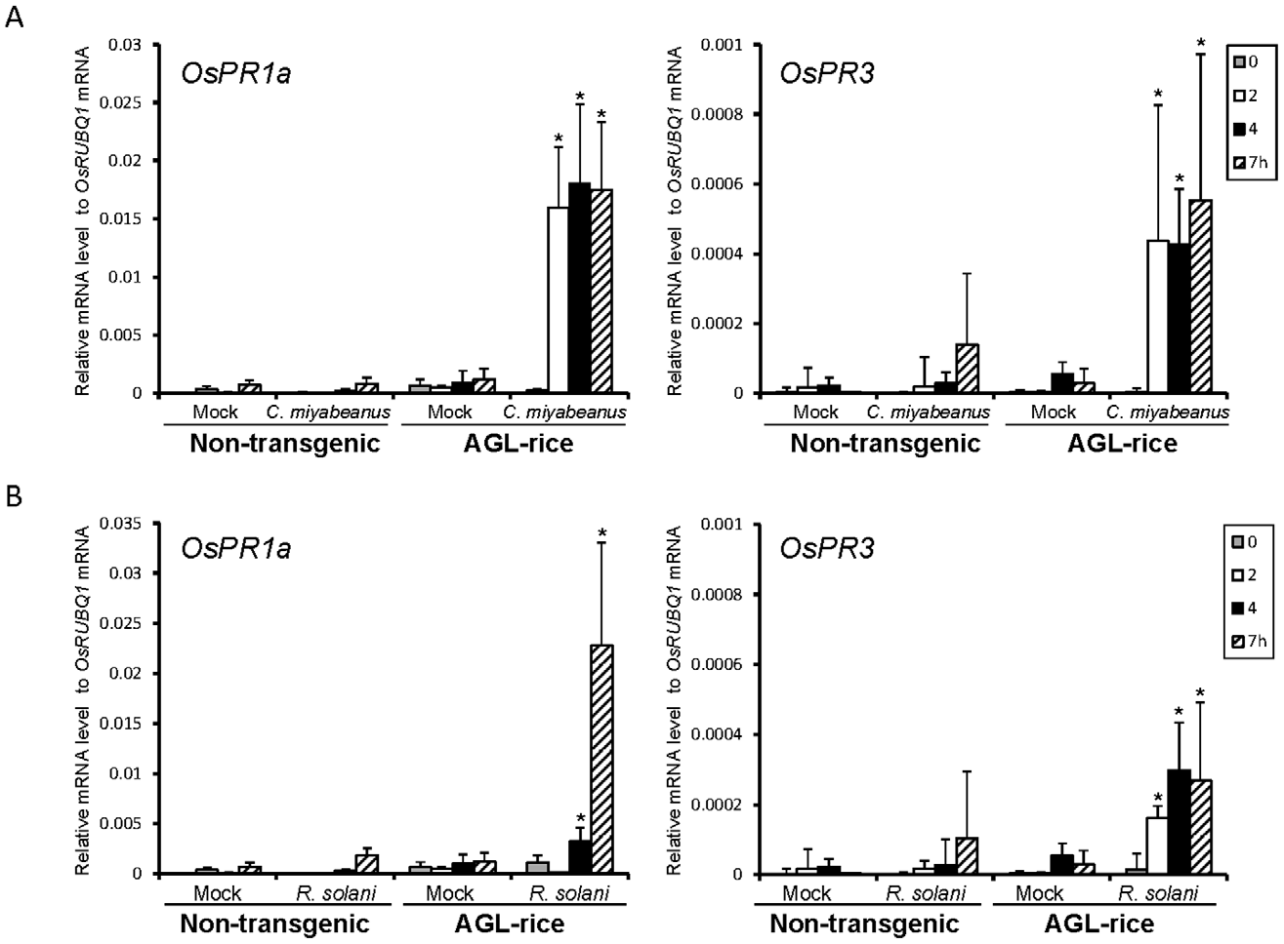
The expression of representative PR genes in *AGL*-expressing rice inoculated with *C. miyabeanus* and *R. solani*. (A) *C. miyabeanus*, and (B) *R. solani*. The expression of *OsPR1a* and *OsPR3* were analyzed. For a control, plants were inoculated with distilled water (mock). The gene expression levels relative to the *OsRUBQ1* gene were analyzed using qRT-PCR at 0, 2, 4, and 7 hpi. The expression of the PR genes was rapidly induced through *ags1* inoculation. The results represent the mean ± SD from eight independent experiments. Asterisks indicate significant differences against the control (mock) at the same time points by the Dunnett's test at *P*<0.05.

We further analyzed whether challenges with *C. miyabeanus* and *R. solani* evoked rapid defense responses in the AGL-rice plants. Expression of *OsPR1* and *OsPR3* was significantly upregulated in the *C. miyabeanus*- and *R. solani*-inoculated leaf sheath cells before 4 hpi, at least 12 h prior to the fungal invasion into the rice cells (Figure 8). Therefore, as in *M. oryzae*, accumulation of α-1,3-glucan on the cell wall interfered with activation of rice defense responses in *C. miyabeanus* and *R. solani*, indicating the relevance of α-1,3-glucan in successful infection in multiple fungal pathogens.

## Discussion

Fungal pathogens have mechanisms for protection against host innate immunity during infection. However, these mechanisms have not been well characterized. We have previously demonstrated that the ascomycete rice pathogen *M. oryzae* synthesizes α-1,3-glucan, an nondegradable polysaccharide in plants, to mask the cell wall surface during infection [10]. In this study, we have revealed that α-1,3-glucan is indispensable for successful infection in *M. oryzae* (Figure 1). Furthermore, we have shown that α-1,3-glucanase-secreting rice showed strong resistance against *M. oryzae* (Figure 4). Considering that α-1,3-glucanase treatment did not prevent the development of the infectious structure (Figure S8) but increased the sensitivity to chitinase (Figure S4), α-1,3-glucan likely plays an important role in the protection of the fungal cell wall from antifungal agents secreted by plants during infection. Because the deficiency or damage of α-1,3-glucan on the fungal cell walls was accompanied by rapid activation of defense-related genes in the inoculated host plants (Figures 3 and 5), the biological significance of the surface protection with α-1,3-glucan would be at least in delaying the release of PAMPs and host defense responses.

The plant cuticular layer has long been believed to be the first barrier against microbial attacks [18]. Therefore, it is considered that fungi are first exposed to antifungal enzymes/compounds present in the apoplast after breaching the cuticular layer, and then the recognition of the fungal PAMPs occurs via the host PRRs located on the cell membranes [19], [20]. Many studies have focused on plant defense responses, including the activation of signaling networks and fungal counter-defense responses upon PRR recognition [20]; however, little is known about the plant-fungus interactions in the early stages of infection. In this report, we have shown that the lack of the surface α-1,3-glucan on *M. oryzae* results in the destruction of fungi on the inoculated leaf sheaths (Figure 2 and S3A). Notably, *ags1* was destroyed before penetration into the apoplast. This destruction is unlikely to occur as a result of the direct contact of the fungal cells with the antifungal agents in the apoplast because the rice cuticular wax layer covered the plant surface underneath the *M. oryzae* germ tubes (Figure S9). Therefore, it is reasonable to suppose that rice plants have an unidentified ‘antifungal barrier’ on the cuticular layer and α-1,3-glucan is one of the factors that enables the pathogenic fungi to overcome this barrier. This barrier is likely composed of antifungal agent(s) secreted onto the plant surfaces. Because the *ags1* mutant lacking α-1,3-glucan was not damaged on the heat-killed rice cells (Figure S2B), this barrier is heat-labile. Plausible candidates for heat-labile components of the barrier are the antifungal enzymes, such as chitinase, which are expressed at basal levels and can damage the fungal cell wall. Other antifungal agents might also be orchestrated to cause detrimental damage to the fungal cells. Alternatively or in addition, *M. oryzae* lacking α-1,3-glucan might release something that can elicit plants' defense responses. Whichever the case, without the surface α-1,3-glucan, the fungal PAMPs would easily be displayed to the host PRRs and the subsequent activation of innate immune signaling could strengthen the integrated defense activity in the host much earlier than that occurring in the normal infection, as observed in the enhanced and rapid expression of the PR genes (Figure 3 and Figure 5). Overall, the pathogen first encounters immune defenses on the cuticular layer of the host plant, and masking the cell wall with α-1,3-glucan is prerequisite for the fungi to pass the hosts' first line of defense.

In this study, we showed that α-1,3-glucan is inducibly accumulated on the cell walls and concealed chitin in *C. miyabeanus* and *R. solani* (Figures 6 and 7), as in *M. oryzae* [10]. The induction occurred when these fungi were grown on the leaf surfaces but not in the synthetic medium, indicating that these fungi are capable of sensing factors derived from plant leaves. In *M. oryzae*, the accumulation of α-1,3-glucan is induced in response to the plant cuticular wax component 1,16-hexadecanediol [10]. However, 1,16-hexadecanediol did not induce α-1,3-glucan accumulation in *C. miyabeanus* or *R. solani* (data not shown); therefore, these fungi detect some other plant component. Although the accumulated α-1,3-glucan concealed chitin in response to plant cues, complete masking throughout infection may not be possible; actually, we observed chitin, albeit very infrequently, at the tips of newly developed infectious hyphae. Plant PRRs might have a chance to recognize chitin elicitors derived from such a locally exposed portion of the hyphae. Unlike *M. oryzae*, the accumulated α-1,3-glucan did not conceal β-1,3-glucan in the cell walls of infectious hyphae in *C. miyabeanus* or *R. solani* (Figures 6 and 7). β-glucans (e.g., branched β-1,3-glucans) are also known to elicit defense responses in plants [21]. Nevertheless, defense responses were not rapidly evoked in the wild type rice challenged by these fungi (Figure 8). Therefore, *C. miyabeanus* and *R. solani* possibly have some mechanism(s) by which the β-glucan recognition in plants and/or subsequent activation of defense responses is compromised.

We have shown that the infection-specific masking of chitin by α-1,3-glucan is conserved among *M. oryzae*, *C. miyabeanus* and *R. solani*. Importantly, the three fungi are mutually distinct in terms of phylogeny, infectious lifestyle and host ranges. *M. oryzae* is a hemibiotrophic pathogen; upon infection, the fungus invades the plant cells biotrophically and subsequently grows necrotrophically [22]. In contrast, *C. miyabeanus* and *R. solani* are necrotrophic pathogens that damage the plant cells with phytotoxins, which are considered to be essential factors for the fungal virulence that help the fungi to obtain nutrients from the damaged host cells [23]–[26]. Phylogenetically, *M. oryzae* and *C. miyabeanus* belong to different orders and are distant within ascomycetes [27], whereas *R. solani* is a basidiomycete. Considering the fact that no genes have been annotated as putative α-1,3-glucanases in plants, it is likely that many fungal plant pathogens have evolved to utilize α-1,3-glucan to conceal the cell-wall PAMPs and in some cases, to maintain the infectious structures. In fact, putative α-1,3-glucan synthase genes are not only present in the currently available genome sequences of monocot cereal fungal pathogens, the anthracnose fungus *Colletotrichum graminicola*, the blotch fungus *Mycosphaerella graminicola* and the rust fungus *Puccinia graminis*, but also in the genome sequences of polyphagous fungal pathogens, such as the white mold fungus *Sclerotinia sclerotiorum* and the gray mold fungus *Botrytis cinerea*. Concealment of the cell wall surfaces accompanied with the non-infectious to infectious phase transition is also known in several human fungal pathogens [2], [28]. In these fungi, various host cues trigger cell wall concealment, which results in reduced stimulation of the host innate immunity [2], [28]. α-1,3-glucan is one of the fungal factors with a concealing function in human pathogens; for example, in the dimorphic *Histoplasma capsulatum*, the α-1,3-glucan layer shields cell wall β-glucan, a major immune stimulatory component [29]. Together with our results, it is very likely that masking the cell wall surface in response to host cues is a common evasion strategy against the host innate immune defenses among animal and plant pathogenic fungi.

Our study suggested the relevance of cell wall α-1,3-glucan for fungal infection. Because many fungal species could potentially utilize the same or similar stealth infection strategies, targeting the fungal α-1,3-glucan, for example, by conferring α-1,3-glucanase activity to crop plants or applying an inhibitor of the fungal α-1,3-glucan synthase might provide a versatile strategy for the prevention of a wide variety of fungal diseases in important crops. Although the detailed mechanism is still remains to be solved, the fact is that the removal of the surface α-1,3-glucan rapidly activates the defenses responses of the host plant against the fungal pathogens prior to the fungal invasion, resulting in the inhibition of pathogen infection. Thus, in addition to transgenic or agrochemical approaches, the employment of α-1,3-glucanase-producing microbes as biological control agents is also a potential sustainable crop protection technique against fungal pathogens in which cell wall PAMPs are concealed with α-1,3-glucan.

The current approaches for controlling fungal diseases include the classical breeding of resistance traits, application of fungicides, use of plant health-strengthening chemicals and the generation of transgenic disease-resistant crop plants [30]. However, only limited crops are available for classical resistance breeding and the use of chemicals might cause environmental problems. The generation of transgenic crop plants has been focused on the enhancement of plant components and the responses involved in the plant defense mechanisms as well as on the interference with the functions of pathogens' effectors or virulence factors [30], [31]. The results of our study provide an additional option for crop protection approaches: the activation of plant innate immunity via the exposure of the fungal cell wall PAMPs. Overcoming this fungal stealth infection strategy could potentially confer resistance to a broad spectrum of fungal diseases in crop plants.

## Materials and Methods

### Fungal strains, growth media, conditions and molecular manipulations

The fungal strains are described in Table S2. The *Magnaporthe oryzae* strains were grown on oatmeal medium [32], and the *Cochliobolus miyabeanus* and *Rhizoctonia solani* strains were grown on PDA medium (3.9% potato dextrose broth [BD Difco], 1.6% agar) at 25°C under constant light for 10 days. The transformation, molecular manipulations and storage of the fungal strains were performed as described [10], [32].

### Generation of the *ags1* mutant

The construction of the gene-replacement cassette KOAGS1 is described in Figure S1A. The endogenous *MoAGS1* (MGG_09639; GenBank XM_364794), a single-copy gene encoding α-1,3-glucan synthase in *M. oryzae*, was replaced with KOAGS1 to generate *ags1*. The *MoAGS1* coding region, along with its putative promoter region, was amplified with the AGS1-F1/R3 primer pair and was co-transformed with pAdh5 [32] into *ags1* to generate the *ags1* ^+*MoAGS1*^ strain. The resulting mutants were confirmed using Southern blot analysis (Figure S1B). The primers used for generation of *ags1* are listed in Table S3.

### Inoculation assays

To test the pathogenicity of the *M. oryzae* strains with a Guy11-background, conidial suspensions (1×10^5^ conidia ml^−1^ in distilled water) were sprayed onto rice (*Oryza sativa* L. *japonica* cv. Lijiangxintuanheigu: LTH) and barley (*Hordeum vulgare* L. cv. Golden promise) plants grown in pots. To test the disease-resistance of the rice plants with a Nipponbare BL2 (*Oryza sativa* L. *japonica* cv. Nipponbare BL no. 2) background, conidial suspensions (1×10^5^ conidia ml^−1^ in distilled water) of *M. oryzae* Ina86-137 and *C. miyabeanus* were spray-inoculated onto the detached rice leaves, and *R. solani* mycelia were placed onto the detached leaves or rubbed onto the cut end of the rice culms as described [33]. The inoculated leaves were incubated on moistened filter paper in plastic containers under constant light at 25°C for *M. oryzae* and *C. miyabeanus* and at 30°C for *R. solani*. The fully expanded uppermost leaves and culms of four-leaf rice seedlings were used for the assays.

### Observation of the fungal infectious structures

The infectious structures of *M. oryzae* were observed as described [10]. The *C. miyabeanus* and *R. solani* mycelia were incubated on glass coverslips with distilled water for 16 h at 25°C. To observe the *C. miyabeanus* infectious hyphae, 50-µl aliquot of the conidial suspensions (1×10^4^ conidia ml^−1^ in distilled water) were placed on rice leaf sheaths and incubated for 16 h at 25°C. To observe the *R. solani* infectious hyphae, the cut end of the rice leaf sheath was rubbed with the fungal mycelia and incubated for 72 h at 25°C. For enzymatic digestion of α-1,3-glucan, fungal cells were fixed with 3% (v/v) formaldehyde in 90% (v/v) ethanol on slide glasses [10] and incubated with 10 µg/µl purified α-1,3-glucanase [34] in PBS buffer (137 mM NaCl, 2.7 mM KCl, 8.1 mM Na_2_HPO_4_, 1.5 mM KH_2_PO_4_, pH 7.4) for 6 h at room temperature. Immunofluorescent labeling and detection of the fungal cell wall polysaccharides were performed as described [10]. Briefly, α-1,3-glucan and β-1,3-glucan were detected with fluorophore-labeled antibodies (IgMg MOPC-104E, Sigma and Monoclonal antibody to (1→3)- β-1,3-glucan (Mouse IgG Kappa Light), Biosupplies, respectively) and chitin with fluorophore-labeled lectin (WGA-Alexa532, Sigma).

### Fungal sensitivity assay to cell wall digestive enzymes

Sensitivity assay of appressorium-forming *M. oryzae* conidia to cell wall digestive enzymes was performed as described [10]. Briefly, appressorium-forming conidia incubated on glass cover slips for 16 h at 25°C were treated with 15 µg/ml of purified chitinase A alone [15] or an enzyme mixture containing 15 µg/ml of purified chitinase A [15] and 10 µg/ml purified α-1,3-glucanase [34] for 4 h at 25°C. To test the inhibitory effect of α-1,3-glucanase on infectious structure development in *M. oryzae* and *C. miyabeanus*, 5 µl of 90 µg/ml purified α-1,3-glucanase [34] in PBS buffer were added to 25 µl of conidial suspension (1×10^5^ conidia ml^−1^ in distilled water). The conidial suspension containing α-1,3-glucanase (final concentration, 15 µg/ml) was incubated on glass coverslips for 24 h at 25°C.

### Quantitative reverse transcription-polymerase chain reaction (qRT–PCR) analysis of rice genes

The rice cultivar LTH was used for the inoculation of *M. oryzae* strains and rice lines with Nipponbare BL2 background were used for *C. miyabeanus* and *R. solani*. The rice plants were grown in pots placed in a greenhouse. *M. oryzae*, *C. miyabeanus* conidial suspensions (5×10^5^ conidia ml^−1^ in distilled water), or *R. solani* mycelial suspension (1×10^6^ cells ml^−1^ in distilled water) were placed on rice sheaths using a syringe and incubated at 25°C. At appropriate intervals, total RNA was extracted from the inoculated leaf sheaths and analyzed for the expression of *OsPR1a*, *OsPR3* and *PBZ1*. The *OsRUBQ1* gene encoding polyubiquitin was used as an internal control. The extraction of total RNA from rice plants, cDNA synthesis and qRT-PCR analysis were performed as described [10]. The primers used for the qRT-PCR analysis were designed to amplify approximately 300-bp sequences of the corresponding genes listed in Table S4.

### Transgenic rice expressing the bacterial α-1,3-glucanase

Nipponbare BL2 was transformed with pBI333-EN4-agl carrying the *B. circulans AGL* gene [34] as described [35], [36]. The structure of pBI333-EN4-agl is described in Figure S1. The expression of the *AGL* gene was confirmed using RT-PCR with the RT-AGL-F/R primer pair. The Agl protein was detected using western blot analysis with the rabbit polyclonal antibody raised against the purified Agl protein (Agl-antibody) as the primary antibody and the horseradish peroxidase-conjugate anti-rabbit IgG mouse antibody (GE Healthcare) as the secondary antibody. The enzymatic activity of the Agl protein produced in rice was assayed as described in the subsection ‘Enzyme activity assays’. The primers used for generation of the transgenic rice are listed in Tables S3.

### Extraction of plant proteins

The total protein fraction was obtained by extraction from culms and leaf sheaths of four-leaf rice seedlings with PBS buffer containing a protease inhibitor cocktail (Roche Applied Science; one tablet in 50 ml of PBS) followed by filter-concentration with the Amicon Centriplus YM-3 cup (Millipore). The extracellular (apoplastic and cell wall) protein fraction was prepared as follows: the rice plants were vacuum-infiltrated for 30 min in 50 ml of Tris-MES extraction buffer (containing one tablet of the protease inhibitor cocktail in 50 ml of 1 M NaCl, 30 mM Tris-MES, pH 6.5) followed by centrifugation at 4,000 *g* for 10 min in 15 ml tubes (Corning). Small compounds (<3 kDa), including chlorophylls in the effluent, were removed by filter concentration in the Amicon cup. The remains from the Amicon cup were precipitated with ammonium sulfate (>70% saturation). The resultant precipitate was dissolved in PBS buffer. The protein concentration was determined using the Pierce BCA protein assay reagent (Thermo Fisher Scientific). The activities of the marker enzymes for subcellular fractions were determined as described in the Supplementary methods and summarized in Table S5.

### Transmission electron microscopy (TEM)

Inoculated leaf sheaths were stripped 25 h after inoculation, dissected into approximately 1×2 mm sections in 2–5% glutaraldehyde in 0.067 M potassium phosphate buffer at pH 7.2 and fixed for 12 h at 4°C; postfixation with 1% osmium tetroxide was performed in the same buffer for 2 h at 24°C as reported [37]. The tissues were dehydrated in acetone, transferred to propylene oxide and embedded in a plastic resin mixture of Araldite-Epon. Ultrathin sections were cut using a diamond knife on a Du Pont-Sorvall MT 6000 ultramicrotome, collected on 300×75 mesh Formvar-coated grids, stained with saturated uranyl acetate in 50% ethanol for 10 min and then stained with lead citrate for 10 min. A Hitachi H-7000 transmission electron microscope (Hitachi) was used for observation of the samples.

### Enzyme activity assays

The activity of the α-1,3-glucanase in the intracellular and extracellular fractions of rice plants was determined as described [15], except that the Glucose (GO) assay kit (Sigma) was used to detect the released glucose. The marker enzymes for the intracellular and the extracellular components are listed in Table S4. The presence of nuclear membranes was determined by 5′ nucleotidase activity, which was measured in a coupled enzyme assay as described [38] with modifications. Fifty microliters of each sample was mixed with 450 µl of the reaction solution (1 mM MgCl_2_, 125 mM NaCl, 0.1 mM AMP in 30 mM Tris-MES, pH 8.0) at 37°C for 10 min, followed by the addition of 5 units of adenosine deaminase (Sigma); the inosines released from adenosine were measured spectrophotometrically at 260 nm per mg of protein. The fumarase and cytochrome c oxidase activities were determined as described [39], [40]. The chlorophyll content and the fructose-1,6-bisphosphatase activity were measured as described [40], [41]. The activities of antimycin A-NADH-cytochrome *c* reductase, a marker enzyme for the endoplasmic reticulum, and latent UDPase were determined as described [42]. The activity of α-mannosidase was determined as described [43]. The phosphoenolpyruvate carboxylase activity was determined as described [44]. The activity of cytosolic fructose-1,6-bisphosphatase was determined in a coupled assay as described [41]. The activity of P-type ATPase was determined as described [45], [46]. The activity of pectinesterase was assayed as described [47].

## Supporting Information

Figure S1 Targeted gene replacement of *MoAGS1* in *Magnaporthe oryzae*. (**A**) Construct for the *MoAGS1* gene replacement. The AGS1-F1/R1 and AGS1-F2/R2 primer pairs were used to amplify 1.5-kb upstream and downstream flanking regions of *MoAGS1*, with additional tag sequences identical to 12 bp at the 5′ end and 10 bp at the 3′ end of the *BAR* gene (GenBank X17220). The *BAR* gene was amplified with the BAR-F/R primer pair from pCAMBIA-Bar1 [34]. KOAGS1 was generated by amplifying a mixture of the *MoAGS1* flanking regions and the *BAR* gene with the AGS1-F1/R2 primer pair. The open boxes indicate the selectable marker gene *BAR* (upper) and the *MoAGS1* gene (lower). The black boxes indicate the homologous 5′- and 3′-flanking regions of the *MoAGS1* gene. The arrowheads show the positions and directions of the PCR primers. The bars (‘Bar’ and ‘AGS1int’) indicate the probes used for the Southern hybridization analyses. E: *Eco*RI sites. Scale bar = 1 kb. (**B**) The Southern hybridization analyses of the *ags1* deletion mutant. The *Eco*RI-digested genomic DNA of the wild-type strain Guy11 (WT), the *ags1* mutant (*ags1*) and the *ags1* mutant containing the wild-type *MoAGS1* gene (*ags1* ^+*MoAGS1*^) were hybridized with the AGS1int (upper panel) and Bar (lower panel) probes. The AGS1int probe hybridized to a 5.6-kb fragment in the WT and *ags1* ^+*MoAGS1*^, but not in *ags1*. The Bar probe hybridized to a 2.3-kb fragment in *ags1* and *ags1* ^+*MoAGS1*^, but not in WT.
(EPS)

Figure S2 Immunofluorescent labeling of polysaccharides in the *ags1* cell wall. (**A**) Immunofluorescent labeling of α-1,3-glucan in the cell wall of the infectious structures of *M. oryzae*. The fungal conidia were incubated on glass coverslips for 24 h for the development of appressoria. The α-1,3-glucan was detected in the WT and *ags1* ^+*MoAGS1*^, but not in *ags1*. Over 300 conidia were observed for each strain and representative images are presented. BF: bright-field optics. Scale bar = 20 µm. (**B**) The fluorescent detection of major cell wall polysaccharides of *M. oryzae* infectious hyphae. The fungal conidia were incubated on heat-killed rice leaf sheath cells for 24 h to develop the infectious hyphae. α- and β-1,3-glucan were detected using specific primary antibodies and fluorophore-conjugated secondary antibodies, and chitin was detected with fluorophore-conjugated wheat germ agglutinin. α-1,3-glucan was detected on the infectious hyphae of WT and *ags1* ^+*MoAGS1*^, but not on those of *ags1*. Note that α-1,3-glucan was observed on incipient appressoria [10] but not on melanized appressoria. β-1,3-glucan and chitin were detected only on the infectious hyphae of *ags1*. BF, bright-field optics; AP, appressorium; IH, infectious hyphae. More than 100 infectious hyphae were observed for each strain and representative images are presented. Scale bar = 20 µm.
(EPS)

Figure S3 The inoculation assays of *ags1* on live susceptible barley plants. (**A**) The degradation of *ags1* cells on live barley leaf sheath cells. At 24 hpi, appressoria were observed with the wild-type *M. oryzae* (WT), whereas the *ags1* cells were destroyed. Observations (>50 conidia for each strain at one time) were repeated 10 times, and representative images are presented. (**B**) Spray inoculation assay. The uppermost fully expanded leaves from 2-week-old barley seedlings were inoculated with WT, *ags1*, or *ags1* ^+*MoAGS1*^ *M. oryzae* conidial suspensions (1×10^5^ conidia ml^−1^). A leaf inoculated with distilled water (mock) served as a control. Typical blast lesions were observed on the leaves of plants inoculated with WT and *ags1* ^+*MoAGS1*^, but not *ags1, M. oryzae* at 5 dpi. The inoculation test was repeated at least 10 times, and representative images are presented.
(EPS)

Figure S4 Cell wall susceptibility against chitinase. Conidia of the wild type (WT), *ags1* and *ags1* ^+*MoAGS1*^ were incubated on glass coverslips with 50 µM 1,16-hexadecanediol for 16 h to induce α-1,3-glucan accumulation. Appressoria-forming conidia were incubated with chitinase (15 µg/ml) alone or both chitinase (15 µg/ml) and α-1,3-glucanase (10 µg/ml) for 4 h. Results are presented as the percentage of germ tubes that were lysed after 4 h incubation. Results represent the mean ± SD of at least three experiments. Asterisks indicate no statistical differences by Tukey's test (*p*>0.1).
(EPS)

Figure S5 The expression of α-1,3-glucanase in transformed rice plants. (**A**) The binary vector pBI333-EN4-agl was used for expression of the *AGL* gene in rice plants. A 3.8-kb DNA fragment encoding the *B. circulans AGL* gene was amplified from pET22-agl [15] using the AGL-F/R primer pair and cloned into a binary vector pBI333 [13] to create pBI333-EN4-agl. LB and RB, T-DNA *left* and *right* borders; P_35S_, the promoter for cauliflower mosaic virus (CaMV) 35S transcript; T_CaMV_, CaMV terminator; EN4, the enhanced CaMV 35S promoter; T_NOS_, terminator of the nopaline synthase gene; *AGL*, the *B. circulans* α-1,3-glucanase gene; *HPH*, the hygromycin phosphotransferase gene. (**B**) RT-PCR analysis of the cDNA from T_1_ transgenic rice leaves (AGL-rice lines #2-1, #2-7, #3-1) and the non-transgenic (NT) Nipponbare BL2 rice leaves. The transcripts from *AGL* (upper panel) and *OsRUBQ1* (control: lower panel) in the transgenic rice plants were confirmed. (**C**) Western blot analysis. The specific binding of the Agl antibody to the purified recombinant Agl protein (135 kDa) produced in *Escherichia coli* and total proteins extracted from the transgenic rice leaves (AGL-rice lines #2-1, #2-7, #3-1), but not to proteins from the NT rice, were observed.
(EPS)

Figure S6 The growth of the transgenic rice expressing the Agl protein. Transgenic rice plants in the T_2_ generation expressing the Agl protein (AGL-rice #3-1: right panel) showed growth comparable to Nipponbare BL2 (non-transgenic: left panel) and transgenic rice exhibiting hygromycin resistance but expressing neither the *AGL* gene transcription nor the Agl protein (non *AGL*-expressing #5-1: center panel).
(EPS)

Figure S7 Infectious structure development on rice leaf sheath. The *M. oryzae* wild-type strain Ina86-137 was inoculated on leaf sheaths of non-transgenic Nipponbare BL2 (Non-transgenic) and the *AGL*-expressing transgenic rice (AGL-rice). The early stage of cell death indicated by cell browning was induced in the rice cells penetrated by *M. oryzae*. Representative images of 200 fungal cells from 5 independent experiments are presented. AP, appressorium; PP, penetration peg; IH, infectious hyphae; CO, conidia. Scale bar = 10 µm. Photos were taken at 48 hpi.
(EPS)

Figure S8 Effect of α-1,3-glucanase on fungal growth. *M. oryzae* and *C. miyabeanus* were incubated with/without α-1,3-glucanase (10 µg/ml) on glass coverslips for 24 h. More than 100 cells for each were observed and representative images are presented. α-1,3-glucanase showed no inhibitory effect on the development of infectious structures. Scale bar = 10 µm.
(EPS)

Figure S9 Transmission electron micrographs of the infectious structures of *M. oryzae*. Upper panel: cross section of the infectious structures. The fungus penetrates the rice epidermal cell via the appressorium, which is formed on the tip of the germ tube. Lower panel: close-up of the germ tube. A cuticle layer covers the host cell wall underneath the germ tube. GT, germ tube; SEP, septum; Ap, appressorium; IH, infectious hyphae; PP, penetration peg; HCW, host cell wall; EC, epidermal cell; Cu, cuticular layer.
(EPS)

Table S1 No defects were observed in appressorium formation on the inductive surfaces or in the development of infectious hyphae in cells of heat-killed rice or onion epidermis in the *M. oryzae* mutant lacking α-1,3-glucan.
(DOCX)

Table S2 List of fungal strains used in this study.
(DOCX)

Table S3 List of primers used in this study.
(DOCX)

Table S4 List of qRT-PCR primers used in this study.
(DOCX)

Table S5 The activities of marker enzymes in fractions of total and extracellular protein from rice plants showed that contamination of the intracellular proteins was very little in the extracellular protein fraction.
(DOCX)
