# Supplementary material for: Surface α-1,3-Glucan Facilitates Fungal Stealth Infection by Interfering with Innate Immunity in Plants
Source: PLoS Pathog. 2012 Aug 23;8(8):e1002882. doi: 10.1371/journal.ppat.1002882 (PMC3426526; doi:10.1371/journal.ppat.1002882)
Supplement: Table S1 — No defects were observed in appressorium formation on the inductive surfaces or in the development of infectious hyphae in cells of heat-killed rice or onion epidermis in the M. oryzae mutant lacking α-1,3-glucan. (DOCX) [file ppat.1002882.s010.docx]

**Table S1. Comparisons of fungal development between *M. oryzae* strains**

|  |  | Guy11 |  | *ags1* |  | *ags1*^+^*^MoAGS1^* |
| --- | --- | --- | --- | --- | --- | --- |
| Colony size (cm) ^1^ |  | 3.9 ± 0.1 |  | 3.9 ± 0.1 |  | 4.0 ± 0.1 |
| Germination rates (%) ^2^ |  | 74.8 ± 3.0 |  | 71.1 ± 1.5 |  | 69.6 ± 5.5 |
| Appressorium formation rates (%) ^3^ |  | 97.5 ± 2.0 |  | 97.9 ± 1.9 |  | 97.5 ± 1.2 |
| Penetration rates (%) ^4^ |  | 55.8 ± 13.3 |  | 56.7 ± 11.9 |  | 56.8 ± 7.9 |

^1^ Colony size was measured after incubating 8 days at 25 °C.

^2^ Germination rates were determined 1h after incubation started on glass cover slips.

^3^ Percentage of conidia that formed appressoria by 24 h on glass cover slips.

^4^ Percentage of appressoria generated penetration peg by 48h into heat-killed onion epidermis cells.
