# Supplementary material for: Surface α-1,3-Glucan Facilitates Fungal Stealth Infection by Interfering with Innate Immunity in Plants
Source: PLoS Pathog. 2012 Aug 23;8(8):e1002882. doi: 10.1371/journal.ppat.1002882 (PMC3426526; doi:10.1371/journal.ppat.1002882)
Supplement: Table S2 — List of fungal strains used in this study. (DOCX) [file ppat.1002882.s011.docx]

**Table S2. Fungal strains used in this study**

| **Fungal  strains** | | |  | **Brief descriptions** |  | **References or  sources** | |
| --- | --- | --- | --- | --- | --- | --- | --- |
| *Magnaporthe oryzae* | | | | | |  |  |
|  | Guy11 | |  | Wild type, *MAT1-2*, compatible to rice cultivar LTH | |  | Dr. D. Tharreau, INRA |
|  | *ags1* | |  | Δ*MoAGS1* mutant with Guy11 background | |  | This study |
|  | *ags1*^+^*^MoAGS1^* | |  | *ags1* supplemented with the wild-type *MoAGS1* | |  | This study |
|  | Ina86-137 | |  | Wild type, compatible to rice cultivar Nipponbare BL2 | |  | Dr. N. Hayashi, NIAS |
| *Cochliobolus miyabeanus* | | | | | |  |  |
|  | MAFF305425 | |  | Wild type, rice pathogen | |  | NIAS Genebank * |
| *Rhizoctonia solani* | | | | | |  |  |
|  | MAFF305219 |  | | Wild type, AG-1, rice pathogen | |  | NIAS Genebank |

* Genebank, National Institute of Agrobiological Sciences, Tsukuba, Japan (http://www.gene.nias.go.jp)
