# Supplementary material for: Surface α-1,3-Glucan Facilitates Fungal Stealth Infection by Interfering with Innate Immunity in Plants
Source: PLoS Pathog. 2012 Aug 23;8(8):e1002882. doi: 10.1371/journal.ppat.1002882 (PMC3426526; doi:10.1371/journal.ppat.1002882)
Supplement: Table S3 — List of primers used in this study. (DOCX) [file ppat.1002882.s012.docx]

**Table S3. Primers used in this study**

| **Primer name** | **Sequence (5’ to 3’)** |
| --- | --- |
| AGS1-F1  AGS1-R1  AGS1-F2  AGS1-R2  BAR-F  BAR-R  AGS1int-F  AGS1int-R  AGS1-R3  AGL-F  AGL-R  RT-AGL-F  RT-AGL-R | CAGGTGTGGTTCCACAGA  ATGACAGCGAGCCACTGGGGAGATATGGG  Underline indicates the tag sequence identical to the 5’-end of the *BAR* gene  CAGGAGGCCTGGTTCATCCATTTGCTTTC  Underline indicates the tag sequence identical to the 3’-end of the *BAR* gene CTCATTCGTCACACGTTC  GCTCGCTGTCATTTTCGAGA  AGGCCTCCTGGGCACGGTCG  ATCGGCATCATCGTGTACCT  CGCCGATGCTCTCGCTAA  ACACCACGAAAGGTCTAGCA  CTCGAGATGCCAAATCTGACATTAGGC  Underline indicates additional *Xho*I site  GAGCTCTTACTGGATTGTAAGATTAAAGCCATT  Underline indicates additional *Sac*I site  AAAACGTCACGTTCACCA  GTTGAAGGTGGCAGAACC |
