# Supplementary material for: Surface α-1,3-Glucan Facilitates Fungal Stealth Infection by Interfering with Innate Immunity in Plants
Source: PLoS Pathog. 2012 Aug 23;8(8):e1002882. doi: 10.1371/journal.ppat.1002882 (PMC3426526; doi:10.1371/journal.ppat.1002882)
Supplement: Table S4 — List of qRT-PCR primers used in this study. (DOCX) [file ppat.1002882.s013.docx]

**Table S4. qRT-PCR primers used in this study**

| **Target gene**  (GenBank Acc. #) | **Primer name** | **Sequence (5’ to 3’)** |
| --- | --- | --- |
| *OsRUBQ1*  (AK121590: polyubiquitin)  *OsPR1a*  (AF251277)  *OsPR3*  (X87109: chitinase)  *PBZ1* (*OsPR10a*)  (AK071613) | OsRubq1-F  OsRubq1-R  OsPR1a-F  OsPR1a-R  OsPR3-F  OsPR3-R  OsPBZ1-F  OsPBZ1-R | GTGGTGGCCAGTAAGTCCTC GGACACAATGATTAGGGATCA  TCGTATGCTATGCTACGTGTTT CACTAAGCAAATACGGCTGACA  TACTGTGTCCAGAGCTCGCAGTGG  TCTGGTTGTAGCAGTCCAAGTTGG  GAGGAATACTGCCTCTATCC  CTCTCACGGACTCAAACGCCA |
