# Supplementary material for: Surface α-1,3-Glucan Facilitates Fungal Stealth Infection by Interfering with Innate Immunity in Plants
Source: PLoS Pathog. 2012 Aug 23;8(8):e1002882. doi: 10.1371/journal.ppat.1002882 (PMC3426526; doi:10.1371/journal.ppat.1002882)
Supplement: Table S5 — The activities of marker enzymes in fractions of total and extracellular protein from rice plants showed that contamination of the intracellular proteins was very little in the extracellular protein fraction. (DOCX) [file ppat.1002882.s014.docx]

**Table S5. The activities of marker enzymes in fractions of total and extracellular protein from rice plants**

| **Organelles** | **Marker enzymes** | **Enzyme activities in the total protein fraction** | |  | **Enzyme activities in the extracellular protein fraction** | |
| --- | --- | --- | --- | --- | --- | --- |
|  |  | **NT** | **AGL-rice** |  | **NT** | **AGL-rice** |
| Nuclear membrane | 5' Nucleotidase ^1^ | 0.28 ± 0.07 | 0.56 ± 0.30 |  | nd | nd |
| Mitochondria | Fumarase ^2^ | 1.28 ± 0.16 | 3.11 ± 0.56 |  | 0.16 ± 0.23 | 0.09 ± 0.36 |
|  | Cytochrome c oxidase ^3^ | 4.86 ± 0.98 | 6.58 ± 2.51 |  | nd | nd |
| Chloroplast | Chlorophyll ^4^ | nd | nd |  | nd | nd |
|  | Fructose bisphosphatase ^5^ | 23.60 ± 0.20 | 19.27 ± 2.29 |  | nd | nd |
| Endoplasmic reticulum | NADH:cytochrome c reductase ^6^ | 4.86 ± 3.93 | 2.98 ± 0.06 |  | nd | nd |
| Vacuole | Alpha-mannosidase ^7^ | 10.41 ± 0.49 | 11.30 ± 1.14 |  | 1.10 ± 0.43 | 1.07 ± 0.30 |
| Golgi body | Latent UDPase ^8^ | 14.13 ± 9.47 | 8.35 ± 2.22 |  | nd | nd |
| Cytosol | Phosphoenolpyruvate carboxylase ^9^ | 5.90 ± 5.60 | 1.57 ± 0.30 |  | nd | nd |
|  | Cytosolic fructose bisphosphatase ^5^ | 16.45 ± 1.87 | 20.05 ± 0.74 |  | nd | nd |
| Plasma membrane and cell wall | P-type ATPase ^10^ | 22.10 ± 3.19 | 15.92 ± 0.53 |  | 62.92 ± 2.05 | 67.22 ± 3.96 |
|  | Pectinesterase ^11^ | -1.11 ± 1.37 | -0.68 ± 0.22 |  | -6.83 ± 4.55 | -5.75 ± 2.11 |

nd: not detected, NT: non-transgenic Nipponbare-BL2. Low or no activities of marker enzymes for cellular organelles indicated that contamination with the intracellular proteins was minimal in the extracellular protein fraction. All values are the means ± standard deviation (SD) for three independent experiments.

^1^ Increase in absorbance for inosinic acid at 260 nm (ΔA_260nm_・min^-1^・mg^-1^).

^2^ Increase in absorbance for fumarate acid at 230 nm (ΔA_250nm_・min^-1^・mg^-1^).

^3^ Increase in absorbance for oxidized cytochrome c at 550 nm (ΔA_550nm_・min^-1^・mg^-1^).

^4^ Amount of chlorophyll (μ・mg^-1^).

^5^ Increase in absorbance for 6-phosphogluconolactone at 340 nm (ΔA_340nm_・min^-1^・mg^-1^).

^6^ Decrease in absorbance for reduced cytochrome c at 550 nm (ΔA_550nm_・min^-1^・mg^-1^).

^7^ Increase in absorbance for mannan at 405 nm (ΔA_405nm_・min^-1^・mg^-1^).

^8^ Increase in absorbance for UMP at 690 nm (ΔA_690nm_・min^-1^・mg^-1^).

^9^ Increase in absorbance for oxaloacetate at 270 nm (ΔA_270nm_・min^-1^・mg^-1^).

^10^ Increase in absorbance for Pi (as Pi-molybdate-complex) at 860 nm (ΔA_860nm_・min^-1^・mg^-1^).

^11^ Decrease in pH (ΔpH・min^-1^・mg^-1^).
